# Correction: Effects of nurse-led transitional care interventions for patients with heart failure on healthcare utilization: A meta-analysis of randomized controlled trials

**DOI:** 10.1371/journal.pone.0262979

**Published:** 2022-01-19

**Authors:** Minlu Li, Yuan Li, Qingtong Meng, Yinyin Li, Xiaomeng Tian, Ruixia Liu, Jinbo Fang

The affiliations for the first author are incorrect. Minlu Li is not affiliated with both #1 and #2, but #1 only: General Ward of Neurology, West China Hospital/ West China School of Nursing, Sichuan University, Chengdu, Sichuan, China.
