# Supplementary material for: Effects of nurse-led transitional care interventions for patients with heart failure on healthcare utilization: A meta-analysis of randomized controlled trials
Source: PLoS One. 2021 Dec 16;16(12):e0261300. doi: 10.1371/journal.pone.0261300 (PMC8675680; doi:10.1371/journal.pone.0261300)
Supplement: S1 File — (PDF) [file pone.0261300.s002.pdf]

Search strategies for databases

CENTRAL

#1 MeSH descriptor: [Heart Failure] explode all trees

#2 ((cardi* or heart* or myocard*) near (fail* or incompet* or insufficien* or decomp* or weak* or dysfunction))

#3 #1 or #2

#4 MeSH descriptor: [Case Management] explode all trees

#5 MeSH descriptor: [Disease Management] this term only

#6 (manage* near/2 (care? or case? or disease?))

#7 MeSH descriptor: [Continuity of Patient Care] explode all trees

#8 MeSH descriptor: [Ambulatory Care] this term only

#9 MeSH descriptor: [Long-Term Care] explode all trees

#10 MeSH descriptor: [Long-Term Care] explode all trees

#11 (manage* near/2 (care? or case? or disease?))

#12 ((transition* or transfer* or continu* or coordinat* or collaborat* or seamless or integrat* or comprehensive or intermediate or extended or follow-up or postdischarge or post-discharge or posthospital* or post-hospital* or home or home-based or domiciliary or long-term or outpatient or ambulatory) near/3 (care? or service? or intervention?))

#13 (interdisciplinary or multidisciplinary or interprofessional) near/3 (program* or management)

#14 patient near/2 (discharge or follow-up) or (discharge? near/2 plan*) or (case near/2 management) or aftercare* or "after care"

#15 MeSH descriptor: [Monitoring, Ambulatory] explode all trees

#16 MeSH descriptor: [Telemedicine] explode all trees

#17 MeSH descriptor: [Telenursing] explode all trees

#18 ((ambulatory or remote or distan* or outpatient) near/3 monitor*) or ((telemed* or tele-med* or telehealth* or tele-health* or tele-consult* or eHealth or mHealth or “Mobile Health” or telecare* or telenurs* or telemonitor* or teleconsult* or tele-consult* or teleconferenc* or tele-conferenc* or telecommunicat* or telecardiol* or telephon* or telemetry or phone* or messag*)):ti,ab,kw

#19 MeSH descriptor: [Nursing Staff] explode all trees

#20 MeSH descriptor: [Advanced Practice Nursing] explode all trees

#21 MeSH descriptor: [Nurse Practitioners] explode all trees

#22 MeSH descriptor: [Nursing] explode all trees

#23 nurs*:ti

#24 #4 or #5 or #6 or #7 or #8 or #9 or #10 or #11 or #12 or #13 or #14 or #15 or #16 or #17 or #18 or #19 or #20 or #21 or #22 or #23

#25 #3 and #24 with Cochrane Library publication date from Jan 2000 to Jun 2020, in Trials

MEDLINE Ovid

1. exp heart failure/ or ((cardi* or heart* or myocard*) adj2 (fail* or incompet* or insufficien* or decomp* or weak* or dysfunction)).tw.
2. case management/ or disease management/ or (manage* adj2 (care? or case? or disease?)).tw.
3. continuity of patient care/ or aftercare/ or patient discharge/ or transitional care/ or ambulatory care/ or long-term care/ or home Care Services/ or home care services, hospital-based/ or home health nursing/
4. ((transition* or transfer* or continu* or coordinat* or collaborat* or seamless or integrat* or comprehensive or intermediate or extended or follow-up or postdischarge or post-discharge or posthospital* or post-hospital* or home or home-based or domiciliary or long-term or outpatient or ambulatory) adj3 (care? or service? or intervention?)).tw.
5. ((interdisciplinary or multidisciplinary or interprofessional) adj3 (program* or management)).tw.
6. ((patient adj2 (discharge or follow-up)) or (discharge? adj2 plan*) or (case adj2 management) or aftercare* or after care).tw.
7. monitoring, ambulatory/ or telemedicine/ or telenursing/ or (((ambulatory or remote or distan* or outpatient) adj3 monitor*) or (telemed* or telemed* or telehealth* or tele-health* or tele-consult* or eHealth or mHealth or Mobile Health or telecare* or telenurs* or telemonitor* or teleconsult* or tele- consult* or teleconferenc* or tele-conferenc* or telecommunicat* or telecardiol* or telephon* or telemetry or phone* or messag*)).tw.
8. exp nurse practitioners/ or exp advanced practice nursing/ or exp nurses/ or exp nursing staff/

or exp nursing/ or nurs*.ti.

9. 2 or 3 or 4 or 5 or 6 or 7 or 8
10. (randomized controlled trial or controlled clinical trial).pt. or randomized.ab. or placebo.ab. or drug therapy.fs. or randomly.ab. or trial.ab. or groups.ab.
11. exp animals/ not humans.sh.
12. 10 not 11
13. 1 and 9 and 12
14. limit 13 to yr="2000 -Current"

Embase Ovid

1. exp heart failure/ or ((cardi* or heart* or myocard*) adj2 (fail* or incompet* or insufficien* or decomp* or weak* or dysfunction)).tw.
2. case management/ or disease management/ or (manage* adj2 (care? or case? or disease?)).tw.
3. aftercare/ or hospital discharge/ or transitional care/ or ambulatory care/ or long term care/ or home care/
4. ((transition* or transfer* or continu* or coordinat* or collaborat* or seamless or integrat* or comprehensive or intermediate or extended or follow-up or postdischarge or post-discharge or posthospital* or post-hospital* or home or home-based or domiciliary or long-term or outpatient or ambulatory) adj3 (care? or service? or intervention?)).tw.
5. ((interdisciplinary or multidisciplinary or interprofessional) adj3 (program* or management)).tw.
6. ((patient adj2 (discharge or follow-up)) or (discharge? adj2 plan*) or (case adj2 management) or aftercare* or after care).tw.
7. ambulatory monitoring/ or exp telehealth/ or (((ambulatory or remote or distan* or outpatient) adj3 monitor*) or (telemed* or tele-med* or telehealth* or tele-health* or tele-consult* or eHealth or e-Health or mHealth or Mobile Health or telecare* or telenurs* or telemonitor* or teleconsult* or tele-consult* or teleconferenc* or tele-conferenc* or telecommunicat* or telecardiol* or telephon* or telemetry or phone* or messag*)).tw.
8. exp nurse practitioner/ or exp advanced practice nurse/ or exp nurse/ or exp perioperative nursing/ or exp nursing staff/ or exp nursing/ or nurs*.ti.

9. 2 or 3 or 4 or 5 or 6 or 7 or 8
10. (random* or factorial* or crossover* or cross over* or cross-over* or (doubl* adj blind*) or (singl* adj blind*) or assign* or allocat* or volunteer*).tw.
11. crossover procedure/ or double blind procedure/ or randomized controlled trial/ or single blind procedure/
12. 10 or 11
13. (animal/ or nonhuman/) not human/
14. 12 not 13
15. 1 and 9 and 14
16. limit 15 to yr="2000 -Current"

CINAHL EBSCO

S19 S3 AND S17 AND S18 Limiters - Published Date: 20000101-20200631

S18 (MH "Clinical Trials+") OR (PT clinical trial OR TX ((clinic* N1 trial?) OR (singl* N1 blind*) OR (doubl* N1 blind*) OR (singl* N1 mask*) OR (doubl* N1 mask*) OR random*) OR (MH "Placebos") OR TX (placebo* OR assign* OR control* OR allocat*) OR (MH "Crossover Design") OR TX (volunteer* OR crossover* OR cross-over*))

S17 S4 OR S5 OR S6 OR S7 OR S8 OR S9 OR S10 OR S11 OR S12 OR S13 OR S14 OR S15 OR S16

S16 TI (nurse-led or nurse led) OR AB (nurse-led or nurse led)

S15 MH "nursing models"

S14 MH "practice patterns, nurses'"

S13 MH "nurse's role"

S12 MH "nurse practitioners"

S11 (ambulatory or remote or distan* or outpatient) N3 monitor* or (telemed* or tele- med* or telehealth* or tele-health* or tele-consult* or eHealth or mHealth or “Mobile Health” or telecare* or telenurs* or telemonitor* or teleconsult* or tele- consult* or teleconferenc* or tele-conferenc* or telecommunicat* or telecardiol* or telephon* or telemetry or phone* or messag*)

S10 MH "Telehealth+"

S9 patient N2 (discharge OR follow-up) OR (discharge? N2 plan*) OR (case N2 management)

OR aftercare* OR “after care”

S8 (interdisciplinary OR multidisciplinary or interprofessional) N3 (program* OR management)

S7 (transition* OR transfer* OR continu* OR coordinat* OR collaborat* OR seamless OR integrat* OR comprehensive OR intermediate OR extended OR follow-up OR postdischarge OR post-discharge OR posthospital* OR post-hospital* OR home OR home-based OR domiciliary OR long-term OR outpatient OR ambulatory) N3 (care? OR service? OR intervention?)

S6 (MH "Continuity of Patient Care+") OR (MH "After Care") OR (MH "Patient Discharge+") OR (MH "Transitional Care") OR (MH "Ambulatory Care") OR (MH "Long Term Care") OR (MH "Home Health Care")

S5 manage* N2 (care? OR case? OR disease?)

S4 (MH "Case Management") OR (MH "Disease Management")

S3 S1 OR S2

S2 TI ((cardi* or heart* or myocard*) N2 (fail* or incompet* or insufficien* or decomp* or weak* or dysfunction)) or AB ((cardi* or heart* or myocard*) N2 (fail* or incompet* or insufficien* or decomp* or weak* or dysfunction))

S1 MH "heart failure+"
