# Supplementary material for: Effects of nurse-led transitional care interventions for patients with heart failure on healthcare utilization: A meta-analysis of randomized controlled trials
Source: PLoS One. 2021 Dec 16;16(12):e0261300. doi: 10.1371/journal.pone.0261300 (PMC8675680; doi:10.1371/journal.pone.0261300)
Supplement: S2 File — (DOCX) [file pone.0261300.s003.docx]

**The adapted HF Disease Management Scoring Instrument (HF-DMSI)**

| **Intervention Category** | | **Points to be Assigned** |
| --- | --- | --- |
| Recipient Points | | 1=Provider alone  2=Patient alone  3=Patient with some inclusion of caregiver  4=Patient with a caregiver who is central to the intervention |
| Intervention content | |  |
|  | Planning for discharge | 0=No mention of discharge planning  1=Limited to assessment of readiness for discharge (e.g., adequate decongestion)  2=Discharge planning begins at the early stage of hospitalization and continues to the postdischarge stage  3=Comprehensive discharge planning including assessment of personal, social, economic, cultural, religious and other factors that might impact the plan (e.g., cognitive function, health literacy, education level, and postdischarge support) |
|  | Education and counseling aimed at supporting self-care | 0=No mention of education  1=Focus solely on importance of treatment adherence  2=Focus on treatment adherence including some creative methods of improving adherence  3=Focus on surveillance but no mention of actions to be taken in response to symptoms (e.g., no flexible diuretic management)  4=Emphasis on surveillance, management, and evaluation of symptoms in addition to treatment adherence |
|  | Medication management | 0=No mention of medication regimen  1=Some mention of medications (e.g., importance of medication compliance) but not an active part of the intervention. No attempt to intervene with provider to get patients on an evidence-based medication regimen  2=Evidence-based medication regimen advocated but no follow-up with patient or provider to monitor the suggestion  3=Medication regimen monitored, attempt made to get the patient on evidence-based medications, with follow-up monitoring |
|  | Psychosocial support | 0=No mention of a psychosocial (e.g., peer, community) support intervention  1=Psychosocial support mentioned but not integral to intervention  2=Psychosocial support is integral component of intervention |
|  | Surveillance by provider: Remote monitoring | 0=No use of remote monitoring or telehealth  1=Remote monitoring is used in conjunction with other interventions that form the main intervention used  2=Telehealth is essential component of intervention |
| Delivery personnel | | 1=Single generalist nurse  2=Single HF specialist nurse  3=Multidisciplinary nurse-led team |
| Method of communication | | 1=Mechanized via internet or telephone  2=Person-to-person by telephone  3=Face-to-face, individual, or in a group  4=Combined: Face-to-face at least once alone or in a group with individual telephone calls in between meetings |
| Intensity and complexity | |  |
|  | Duration | 1=≤1 mo.  2=≤3 mo.  3=≤6 mo. |
|  | Complexity | 1=Low: single contact with little or no follow-up  2=Moderate: >1 but <4 and/or infrequent contact or contacts of short duration  3=High: multiple contacts of significant duration |
| Environment | | 1=Telephone or internet-based  2=Clinic/outpatient setting  3=Home-based  4=Combination of settings |
| **Total** | |  |
