# Supplementary material for: Effects of nurse-led transitional care interventions for patients with heart failure on healthcare utilization: A meta-analysis of randomized controlled trials
Source: PLoS One. 2021 Dec 16;16(12):e0261300. doi: 10.1371/journal.pone.0261300 (PMC8675680; doi:10.1371/journal.pone.0261300)
Supplement: S3 File — (DOCX) [file pone.0261300.s004.docx]

## Summary characteristics of participants and interventions in the included studies

| Author, Year (Country) | Setting and Sample Size | Participants ^*^ | Interventions | | Outcomes |
| --- | --- | --- | --- | --- | --- |
|  |  |  | Transitional Care Interventions | Comparator |  |
| Aldamiz-Echevarría 2007  (Spain) | Single-center  *N=*279  137 vs. 142 | Mean age: 75.3±11.1 vs. 76.3±9.4; (75.8±10.3)  Male (%): 38.7 vs. 40.1; (39.6)  Severity of HF (%):  NYHA ^†^: NR  LVEF ^‡^: 50.9±16.6 (*N_1_*=130) vs. 48.3±17.6 (*N_2_*=124); (49.6±17.1)  Medication at discharge (%):  ACEI/ARB: 85.4 vs. 82.4  *β-*blockers: 13.1 vs. 37.3 | Intervention category: *Home-visiting program*  Recipient: Patient with some inclusion of family member  Content: (1) Home visits within 15 d of discharge by physicians and nurses, for clinical examination, tests/analyses as required, and adjustment of medication as required (this intervention was not HF-specific, but was intended to reduce readmissions across a range of medical and surgical conditions); (2) additional nursing staff home visits at 2, 5 and 10 d after discharge for education for participants and relatives about HF (basic facts and management, i.e. symptoms, life style, diet and therapy) lasting about 1h per visit; (3) participants received educational manual and a phone number for queries.  Delivery personnel: Single generalist nurse  Communication method: Individual face-to-face  Duration & Complexity: 15 d./multiple contacts of significant duration  Environment: Home-based  Com/ty ^§^: 20 | Usual care (primary care physician). | All-cause readmission  /6 mo. ^¶^  Emergency department visit  /6 mo. |
| Angermann 2012  (Germany) | Multi-center  *N=*715  352 vs. 363 | Mean age: 67.7±12.8 vs. 69.4±11.5; (68.6±12.2)  Male (%): 71 vs. 71; (71)  Severity of HF (%):  NYHA Ⅲ-Ⅳ: 43 vs. 36; (40)  LVEF: 30±8 vs. 30±8; (30±8)  Medication at discharge (%):  ACEI/ARB: 89 vs. 87  *β-*blockers: 81 vs. 79 | Intervention category: *Structured telephone support*  Recipient: Patient with a caregiver who is central to the intervention  Content: (HeartNetCare-HF). (1) Availability of home electronic scales and blood pressure gauges ensured; (2) in-hospital contact between specialist nurse, patient, and relatives to explain the intervention, practice supervision of BP, HR and symptoms, and provide teaching materials and self-monitoring schemes; (3) telephone-based structured monitoring using 19-item questionnaire lasting 10~15 min assessing indicators of worsening HF, other cardiac symptoms, medication, health care utilization, state of mood and general health and well-being (weekly during the first mon, and then individualized according to NYHA class at discharge, i.e., weekly or fortnightly for NYHA III-IV, monthly for NYHA I-II); (4) up titration of HF medication in co-operation with GPs; (5) needs-adjusted specialist care; (6) measures for appropriate education and supervision of interveners to ensure high intervention quality; (7) emergency telephone access to team members and outpatient facilities.  Delivery personnel: Multidisciplinary nurse-led team (HF specialist nurses mainly)  Communication method: Person-to-person by telephone  Duration & Complexity: 6 mo./multiple contacts of significant duration  Environment: Telephone or internet-based  Com/ty: 26 | Usual care typically included treatment plans, comprehensive discharge letters, and appointments with GPs or cardiologists within 7~14 d. | All-cause readmission  /6 mo.  HF readmission  /6 mo. |
| Barth 2001  (USA) | Single-center  *N=*34  17 vs. 17 | Mean age: 78.0±6.9 vs. 72.4±10.0; (75.2±9.5)  Male (%): 59 vs. 35; (47.1)  Severity of HF (%):  NYHA Ⅲ-Ⅳ: NR  LVEF: NR  Medication at discharge (%):  ACEI/ARB: NR  *β-*blockers: NR | Intervention category: *Structured telephone support*  Recipient: Patient alone  Content: (1) Postdischarge phone calls followed a standard approach based on responses to structured questionings about participants' weight and symptoms (swelling, breathlessness, cough, fatigue, and chest pain). The 1st call within 72 h of discharge, 2nd call within 72 h following the 1st call, 2-week interval calls following the 2nd call; (2) additional probing to clarify answers, reinforce teaching, and make referrals as dictated by the patient’s responses.  Delivery personnel: Single generalist nurse (the investigator)  Communication method: Person-to-person by telephone  Duration & Complexity: 2 mo./multiple contacts of significant duration  Environment: Telephone or internet-based  Com/ty: 15 | Usual care (the routine discharge teaching at the time of discharge as per hospital procedure). | Emergency department visit  /2 mo. |
| de Souza 2014  （Brazil） | Two-center  *N=*252  123 vs. 129 | Mean age: 62±14 vs. 63±12; (62±13)  Male (%): 61.0 vs. 64.3; (62.7)  Severity of HF (%):  NYHA Ⅲ-Ⅳ: 53.5 vs. 58.1; (55.9)  LVEF: 29.2±8.2 vs. 30.1±9.5; (29.6±8.9)  Medication at discharge (%):  ACEI/ARB: 69.9 vs. 74.4  *β-*blockers: 74.7 vs. 72.0 | Intervention category: *Home-visiting program*  Recipient: Patient with some inclusion of caregiver  Content: (1) Four home visits, lasting 1 h each (within 10 d of discharge, approx. 30, 60 and 120 d after discharge), including physical examination, knowledge/self-care assessment and adherence to recommendations, medications info, weight control, hydro-saline restriction, physical activity, and annual vaccination, signs and symptoms to watch for and therapeutic strategies, in addition, a clinical cardiologist could be consulted to adjust drugs where necessary; (2) four reinforcement phone calls, about 10 min each to reinforce the info given at the home visit, check use of medications, and clarify any issues; (3) outpatient clinic visit at 180 days follow-up to finalize assessment.  Delivery personnel: Single HF specialist nurse  Communication method: Combined (Face-to-face contact and telephone calls in between meetings)  Duration & Complexity: 6 mo./multiple contacts of significant duration  Environment: Combination of settings (home visit, telephone contact, and clinic visit)  Com/ty: 26 | Standard treatment approach for HF patients, involving medical outpatient visits with no specific management plan. Typically, patients were followed-up by a GP postdischarge. | All-cause readmission  /6 mo.  HF readmission  /6 mo. |
| Domingues 2011  (Brazil) | Single-center  *N=*111  48 vs. 63 | Mean age: 62±12 vs. 63±13; (63±13)  Male (%): 67 vs. 51; (57.7)  Severity of HF (%):  NYHA Ⅲ-Ⅳ: NR  LVEF: 29±8 vs. 29±9; (29±8)  Medication at discharge (%):  ACEI/ARB: NR  *β-*blockers: NR | Intervention category: *Structured telephone support*  Recipient: Patient with some inclusion of caregiver  Content: (In-hospital education and structured telephone support). (1) 3~5 in-hospital visits lasting 45 min each to provide education about self-care information, HF knowledge, pharmacological treatment and mainly non-pharmacological care (the same with the control group); (2) postdischarge systematic telephone contact for a 3-mon period (1 telephone contact per week during the 1st mon, followed by 1 every 15 d in the 2nd and 3rd mon.) aiming at clarifying and reinforcing in-hospitalization instructions and monitoring signs and symptoms of decompensation. The nurse did not interfere with or change the patients’ pharmacological treatment, but recommended seeing the doctor or going to the emergency care unit when there were signs of HF decompensation.  Delivery personnel: Single generalist nurse (study nurse)  Communication method: Person-to-person by telephone  Duration & Complexity: 3 mo./multiple contacts (totaling 8 calls per patient)  Environment: In-patient and telephone-based  Com/ty: 19 | In-hospital education and postdischarge usual care that consisted of the follow-up of the patient at the return appointment at the outpatient clinic without any telephone contact | All-cause readmission  /3 mo.  Emergency department visit  /3 mo. |
| Ducharme 2005  (Canada) | Single-center  *N=*230  115 vs. 115 | Mean age: 68±10 vs. 70±10; (69±10)  Male (%): 73 vs. 71; (72)  Severity of HF (%):  NYHA Ⅲ-Ⅳ: 93 vs. 88; (90.4)  LVEF: 34±14 vs. 35±15; (34.5±14.5)  Medication at discharge (%):  ACEI/ARB: 84 vs. 76  *β-*blockers: 52 vs. 34 | Intervention category: *Multidisciplinary care model*  Recipient: Patient with some inclusion of family member  Content: (Multidisciplinary HF outpatient clinic-based management and telephone follow-up support). (1) Evaluation at clinic within 2 weeks of hospital discharge including history and physical examination, with particular attention paid to possible remediable exacerbating factors; (2) HF clinic provided rapid access to multidisciplinary team as required and the patient could be evaluated both clinically and para-clinically, receive intravenous diuretics if required and be observed for up to 5 h; (3) after baseline evaluation, clinic cardiologists individualized evidence-based treatment plan for participants; (4) 1-on-1 education of the participant and family initiated at the 1st visit with individualized advice on the disease process, symptoms and signs of HF, fluid and sodium intake restrictions, the importance of daily monitoring of body weight and action plans to remedy weight changes, effects of medications and the importance of compliance, and exercise and diet recommendations; (5) the advice given was individualized and complimented with participant diary for daily weight measurement, medication record, clinical notes and appointments, physical activity recommendations, an education booklet and a telephone number for clinic consultation during business hours; (6) individualized dietary assessments by registered dietician, specific pharmaceutical recommendations by pharmacist at baseline; (7) individualized follow-up plan included monthly visits with both a cardiologist and nurse at the clinic; (8) follow-up phone call from nurse within 72 h of hospital discharge and then monthly to evaluate signs of clinical deterioration and adverse effects and participants were referred to clinic cardiologist if required.  Delivery personnel: Multidisciplinary nurse-led team (clinician nurses mainly)  Communication method: Combined (face-to-face contact with individual telephone calls in between meetings)  Duration & Complexity: 6 mo./multiple contacts of significant duration  Environment: Combination of settings  Com/ty: 28 | Standard follow-up with the attending physicians without further direct contact with the research team or the planned intervention. | All-cause readmission  /6 mo.  Emergency department visit  /6 mo.  Length of hospital stay  /6 mo. |
| Dunagan 2005  (USA) | Single-center  *N=*151  76 vs. 75 | Mean age: 70.5±12.7 vs. 69.4±13.9; (69.9±13.3)  Male (%): 41 vs. 47; (43.7)  Severity of HF (%):  NYHA Ⅲ-Ⅳ: 78 vs. 83; (80.1)  LVEF: < 40 (76.2% of participants)  Medication at discharge (%):  ACEI/ARB: 75 vs.67  *β-*blockers: NR | Intervention category: *Structured telephone support*  Recipient: Patient alone  Content: (An educational packet and scheduled telephone follow-up contact). (1) Regularly scheduled telephonic monitoring by trained nurses to promote self-management skills, appropriate diet, and adherence to guideline-based therapy prescribed by PCP (the 1st call within 3 d after hospital discharge and then at least weekly for 2 weeks, and subsequently adjusted based on assessments of patients’ clinical status and self-care abilities); (2) Patients were encouraged to contact program nurses any time they experienced an increase in symptoms or had questions about their disease or treatment and nurses recommended up titration of diuretics or contacted the patient’s physician for instructions if necessary.  Delivery personnel: Single generalist nurse (study nurse)  Communication method: Person-to-person by telephone  Duration & Complexity: 6 mo./infrequent contact  Environment: Telephone or internet-based  Com/ty: 17 | An educational packet and usual care as provided by their PCP. | All-cause readmission  /6 mo.  HF readmission  /6 mo. |
| Kasper 2002  (USA) | Two-centers  *N=*200  102 vs. 98 | Mean age: 60.2±13.8 vs. 63.7±15.0; (61.9±14.5)  Male (%): 64.7 vs. 56.1; (60.5)  Severity of HF (%):  NYHA Ⅲ-Ⅳ: 55.9 vs. 61.2; (58.5)  LVEF: 27.1±13.8 vs. 27.5±13.9; (27.3±13.8)  Medication at discharge (%):  ACEI/ARB: 97.1 vs. 91.9  *β-*blockers: 38.2 vs. 39.8 | Intervention category: *Clinic-based intervention*  Recipient: Patient alone  Content: (1) HF cardiologist designed an individualized treatment plan for each participant before randomization, which included medication, diet and exercise management; (2) "Telephone nurse co-coordinator" phoned participants within 72 h of discharge and then weekly for 1st mon, bi-weekly in the 2nd mon and then monthly (Content of phone calls: set script with problems pursued as clinically indicated. No medication adjustments over phone. Each lasting about 16 min); (3) Monthly follow-up with HF nurses in HF clinic, lasting about 57 min each to implement the therapeutic plan using a pre-specified 55-page algorithm under the directions of the CHF cardiologists; (4) PCP received regular updates from HF nurses and were notified of abnormal lab results; (5) All intervention participants received: pill sorter, medications list, dietary and exercise recommendations list, 24-h telephone contact number and participant educational material; (6) If required and financial resources limited, participants also received: 3 g sodium 'Meals on Wheels' diet, weigh scale, medications, transport to the clinic and a phone; (7) HF cardiologist saw participants at 6 mon.  Delivery personnel: Multidisciplinary nurse-led team (A CHF nurse and a telephone nurse are dominant )  Communication method: Combined (Face-to-face contact and telephone calls in between meetings)  Duration & Complexity: 6 mo./multiple contacts  Environment: Combination of settings  Com/ty: 26 | Usual care (Patients cared for by their PCP. The baseline therapeutic plan designed by the CHF cardiologist was documented in the patient’s chart, without further intervention). | HF readmission  /6 mo. |
| Kwok 2008  (Hong Kong, China) | Two-center  *N=*105  49 vs. 56 | Mean age: 79.5±6.6 vs. 76.8±7.0; (78.0±6.9)  Male (%): 45 vs. 45; (45)  Severity of HF:  NYHA Ⅲ-Ⅳ: NR  LVEF<40%: 18% vs. 30%  Medication at discharge (%):  ACEI/ARB: 59 vs. 55  *β-*blockers: 18 vs. 25 | Intervention category: *Home-visiting program*  Recipient: Patient alone  Content: (Community nurse-supported hospital discharge program). (1) Community nurse (CN) visited participants prior to discharge, to provide health counselling, such as drug compliance, dietary advice and to encourage participants to contact CN via a telephone hotline during office hours when they developed symptoms; (2) home visit by CN within 7 d of discharge, then weekly for 4 weeks, then monthly, to check signs of poorly controlled HF, medications, compliance and give dietary/exercise advice; (3) home care and day care services arranged if social support insufficient; (4) following liaison with geriatrician or cardiologist, CN was able to alter medication, arrange outpatient appointments and clinical admission as appropriate; (5) CN monitored participants refusing further home visits by telephone.  Delivery personnel: Single generalist nurse (community nurse)  Communication method: Face-to-face contact  Duration & Complexity: 6 mo./multiple contacts  Environment: In-patient and home-based  Com/ty: 21 | Usual medical and social care, with follow-up in the hospital outpatient clinics. | All-cause readmission  /6 mo. |
| Laramee 2003  (USA) | Single-center  *N=*287  141 vs. 146 | Mean age: 70.6±11.4 vs. 70.8±12.2; (70.7±11.8)  Male (%): 58 vs. 50; (54)  Severity of HF (%):  NYHA Ⅲ-Ⅳ: 38 vs. 38; (38)  LVEF: <40 (all of participants)  Medication at discharge (%):  ACEI/ARB: 86 vs. 79  *β-*blockers: 65 vs. 61 | Intervention category: *Case management*  Recipient: Patient with some inclusion of family member  Content: (1) Early discharge planning and coordination of care: When patient in the hospital and for the next 12 weeks, the case manager (CM) assisted in the coordination of care by facilitating the discharge plan and obtaining needed consultations from social services, dietary services, and physical/occupational therapy (PT/OT). The CM also facilitated communication in the hospital between the patient and medical staff. Additional services were arranged once the patient returned home. A letter was sent to the PCP that informed them of their patient’s participation in the study and outlined the case management program after the patient discharge, and the PCP received a letter that summarized the patient’s condition and progress in the program at completion of the 12-week study; (2) Individualized and comprehensive patient and family education: Each day in the hospital and with every telephone contact, the CM conducted and then reinforced the guideline-based education including self-care topics with the patient and family. The patient received educational materials, including a HF booklet, weight logs, self-care activities summary sheets, computerized medication lists, and a guide for measuring sodium intake; (3) Patient and/or family members received telephone follow-up and surveillance at 1 to 3 d after discharge and at weeks 1, 2, 3, 4, 6, 8, 10, and 12 (5 to 45 min per call). If symptoms or signs of CHF were detected during a routine telephone call, appropriate triage arranged and additional telephone calls to the patient were prompted. (4) Patients were instructed to contact their physician anytime a change in symptoms occurred. The CM was also available as a resource Mon through Fri during daytime hours; (5) Promotion of optimal CHF medications and medication doses based on consensus guidelines.  Delivery personnel: Single HF specialist nurse (CHF case manager)  Communication method: Combined (Face-to-face contact and telephone calls)  Duration & Complexity: 3 mo./multiple contacts of significant duration  Environment: Combination of settings  Com/ty: 29 | Standard care, typical of a tertiary care hospital, and all conventional treatments requested by the attending physician. | All-cause readmission  /3 mo.  HF readmission  /3 mo. |
| Linné 2006  (Sweden) | Multi-center  *N=*230  122 vs. 108 | Mean age: 70.3(34-89) vs. 70.8(41-88); 70.5(12.8)  Male (%): 65.6 vs. 75.9; (70.4)  Severity of HF (%):  NYHA Ⅲ-Ⅳ: NR  LVEF: <40 (all of participants)  Medication at discharge (%):  ACEI/ARB: 81 vs. 79  *β-*blockers: 55 vs. 43 | Intervention category: *Primarily educational intervention*  Recipient: Patient alone  Content: Patients received additional pre-discharge education by an interactive program on HF general knowledge, its symptoms, treatment and HF self-care, taking 20~30 min. Two weeks postdischarge, patients returned to the hospital and the CD program was repeated.  Delivery personnel: Single generalist nurse  Communication method: N/A  Duration & Complexity: 2weeks./infrequent contact  Environment: Hospital  Com/ty: 13 | Usual care (patients received the standard info prior to discharge) | All-cause readmission  /6 mo. |
| McDonald 2002  (Ireland) | Single-center  *N*=98  51 vs. 47 | Mean age: 70.76±10.37 vs. 70.83±10.69; (70.8±10.47)  Male (%): 62.7 vs. 70.2; (66.3)  Severity of HF (%):  NYHA Ⅲ-Ⅳ: NR  LVEF: 36±12 vs. 38±15; (37±13)  Medication at discharge (%):  ACEI/ARB: NR  *β-*blockers: NR | Intervention category: *Multidisciplinary care model*  Recipient: Patient with some inclusion of caregiver  Content: In addition to routine care, patients systematically received (1) pre-discharge specialist nurse-led education (daily weighing, understanding disease and medication, salt restriction) and 3 or more specialist dietitian consults; (2) post-discharge telephone contact to ascertain clinical status, provide additional education at 3 d after discharge and weekly thereafter until 12 weeks, HF clinic visits at weeks 2 and 6 to check clinical status and further revise key education issues, HF clinic contact should they notice any clinical deterioration, and advice on self-increase of diuretics according to weight criteria.  Delivery personnel: Multidisciplinary nurse-led team (specialist nurse mainly)  Communication method: Combined (Face-to-face contact and telephone calls)  Duration & Complexity: 3 mo./multiple contacts  Environment: Combination of settings  Com/ty: 27 | Routine care (In-hospital: Patients administered HF investigations and optimal medical therapy, and clinical stability criteria met before discharge. Outpatient: Patients were referred back to their PCP). | HF readmission  /3 mo. |
| Naylor 2004  (USA) | Multi-center  *N*=239  118 vs. 121 | Mean age: 76.4±6.9 vs. 75.6±6.5; (76.0±6.7)  Male (%): 40 vs. 44; (42.7)  Severity of HF (%):  NYHA Ⅲ-Ⅳ: NR  LVEF: < 45 (86% of participants)  Medication at discharge (%):  ACEI/ARB: NR  *β-*blockers: NR | Intervention category: *Case management*  Recipient: Patient with some inclusion of caregiver  Content: (1) A standardized orientation and training program provided by a multidisciplinary team to prepare APNs. (2) Use of care management strategies foundational to the Quality Cost Model of APN Transitional Care, including identification of patients’ and caregivers’ goals, individualized plans of care developed and implemented by APNs in collaboration with patients’ physicians, educational and behavioral strategies to address patients’ and caregivers’ learning needs, continuity of care and care coordination across settings, and the use of expert nurses to deliver and manage clinical services to high risk patient groups. (3) APN implementation of an evidence-based protocol, including: APN discharge planning; an initial APN visit within 24 h of index hospital admission and at least daily during the index hospitalization for comprehensive assessment of participants and caregivers, at least 8 APN home visits (1 within 24 h of discharge, weekly during the first month, bimonthly during the second and third months), additional APN visits based on patients’ needs; and APN telephone availability 7 days per week (8 a.m. to 8 p.m., weekdays; 8 a.m. to noon, weekends). (4) If a patient was rehospitalized during the intervention period, the APN resumed daily hospital visits to facilitate the transition from hospital to home. (5) APNs had email/phone access to multidisciplinary team for consultation of cases as required. (6) APNs collaborated with each participants’ physician regarding adjustments in medications and other therapies or worked under specific guidance from physician. (7) Self-management of symptoms was promoted by APNs teaching participants and caregivers about early symptom recognition and effective treatment, such as the use of as-needed diuretics. (8) Taped teaching material was left with participants.  Delivery personnel: Single HF specialist nurse (trained advanced practice nurse)  Communication method: (Face-to-face contact and telephone calls)  Duration & Complexity: 3 mo./multiple contacts  Environment: Combination of settings  Com/ty: 31 | Routine care (including site-specific discharge planning and clinical paths) for the admitting hospital; Standard home agency care if referred, consisting of comprehensive skilled home health services 7 d/week; On-call registered nurse available 24 h/d; 58% of control participants received skilled nursing or physical therapy after index discharge. | All-cause readmission  /6 mo. |
| Negarandeh 2019  (Iran) | Single-center  *N*=80  40 vs. 40 | Age n(%):  45-50: 15(42.9) vs. 14(42.4)  50-60: 8(22.9) vs. 10(30.3)  60-70: 12(34.3) vs. 9(27.3)  Male (%): 60 (*N_1_*=35) vs. 60.6 (*N_2_*=33); (60.3)  Severity of HF (%):  NYHA Ⅲ-Ⅳ: NR  LVEF: NR  Medication at discharge (%):  ACEI/ARB: NR  *β-*blockers: NR | Intervention category: *Structured telephone support*  Recipient: Patient alone  Content: (1) After hospital discharge, participants were followed up using telephone support lasting for 2 mon. During the 1st mon, two 20-min phone calls were made weekly that could vary based on the patients’ needs and educational questions; in the 2nd mon, calls were made once a week. (2) The educational needs of patients were evaluated before and during the follow-up. Intervention included evaluating patient’s self-care status, providing necessary recommendations and education for performing self-care behaviors, follow-up, and reevaluation. Educational contents were developed based on the needs of the participants and the HF self-care behavior questionnaire using the literature review and evidence-based sources. Education was provided interactively and mostly based on the patients’ conditions.  Delivery personnel: Single generalist nurse (trained nurse)  Communication method: Person-to-person by telephone  Duration & Complexity: 2 mo./multiple contacts  Environment: Telephone or internet-based  Com/ty: 16 | Usual care with education provided at the time of discharge. | HF readmission  /2 mo. |
| Nucifora 2006  (Italy) | Single-center  *N*=200  99 vs. 101 | Mean age: 73±9 vs. 73±8; (73±8.5)  Male (%): 62 vs. 62; (62)  Severity of HF (%):  NYHA Ⅲ-Ⅳ: 67 vs. 62; (64)  LVEF: 43±16 vs. 43±19; (43±17.5)  Medication at discharge (%):  ACEI/ARB: 80 vs. 80  *β-*blockers: 14 vs. 11 | Intervention category: *Case management*  Recipient: Patient alone  Content: (1) Pre-discharge intensive education about HF during a half-hour-long face-to-face session, using a teaching booklet (topics: causes of HF, recognition of symptoms of worsening HF, the role of sodium restriction and pharmacological therapy, the importance of fluid and weight control, physical activity and complete abstinence from alcohol and smoking); (2) phone call from nurse 3-5 d post discharge to assess any problems, promote self-management and check compliance, weight and lifestyle issues; (3) participants had telephone access from 8.00 am to 9.00 am, Mon to Fri, and out of hours answering machine; (4) outpatient visits to doctor at 15 d, 1 and 6 mon after discharge, to evaluate test results, physical condition and medicine adherence and make any required changes to drug therapy.  Delivery personnel: Single HF specialist nurse (experienced HF research nurse)  Communication method: Combined (Face-to-face contact with telephone calls in between meetings)  Duration & Complexity: 6 mo./multiple contacts  Environment: Combination of settings  Com/ty: 25 | Pre-existing routine of post-discharge care; i.e., usual care by primary care physician and outpatient visit to doctor at 6 mon after discharge instructions. | All-cause readmission  /6 mo.  Length of hospital stay  /6 mo. |
| Ong 2016  (USA) | Multi-center  *N*=1437  715 vs. 722 | Mean age: 73 (62-84) vs. 74 (63-82); 73.5±5.2  Male (%): 53.8  Severity of HF (%):  NYHA Ⅲ-Ⅳ: 61.2  LVEF: 42.7(41.3-44.3) vs. 43.0(41.6-44.3)  Medication at discharge (%):  ACEI/ARB: 56.6 vs. 54.6  *β-*blockers: 73.2 vs. 76.1 | Intervention category: *Case management*  Recipient: Patient alone  Content: (Predischarge HF education, scheduled telephone coaching, and home telemonitoring). (1) Predischarge HF education by a study nurse, not part of usual care team used a booklet and the ‘teach-back’ method to ensure understanding. Also included demonstration of telemonitoring equipment and the important of monitoring physiological variables; (2) Regularly scheduled telephone coaching, 9 calls scheduled over 6 mon, usually the same call center nurse. The 1st contact within 2-3 d of discharge, then weekly for the 1st month. Subsequent calls were made monthly until the end of the 6-mon study period. Calls were designed to reinforce predischarge education materials; (3) The electronic equipment consisted of a weight scale, and a BP and HR monitor integrated with a device that could display text questions (3 symptom questions) and send simple text responses. Daily results monitored by call center nurses. Readings that exceeded predetermined threshold variables generated a trigger for the nurse to telephone the patient to investigate potential causes and the nurse will give proper advice.  Delivery personnel: Single generalist nurse (study nurse)  Communication method: Mechanized connection and person-to-person by telephone  Duration & Complexity: 6 mo./multiple contacts  Environment: Telephone or internet-based  Com/ty: 20 | Usual care included robust predischarge education and often a postdischarge follow-up telephone call. No additional surveillance was provided. | All-cause readmission  /6 mo. |
| Pugh 2001  (USA) | Multi-center  *N*=58  27 vs. 31 | Mean age: 73±9 vs. 73±8; (73±8.5)  Male (%): 62 vs. 62; (62)  Severity of HF (%):  NYHA Ⅲ-Ⅳ: 67 vs. 62; (64)  LVEF: 43±16 vs. 43±19; (43±17.5)  Medication at discharge (%):  ACEI/ARB: 80 vs. 80  *β-*blockers: 14 vs. 11 | Intervention category: *Case management*  Recipient: Patient with some inclusion of caregiver  Content: (Enhanced discharge planning and longitudinal nursing case management). (1) The CM assessed patients' HF knowledge and started the discharge planning at inpatient recruitment and teaching programs were individualized accordingly. The educational process was initiated by providing a set of study handouts and other written materials and was supplemented with videotapes related to HF and diet; (2) Patients prescribed a flexible diuretic plan were given written instructions for adjusting their diuretics relative to weight gain; (3) Referrals to other members of the multidisciplinary team were made by the CM as indicated. The need for home care or other outpatient services was evaluated and arranged as necessary; (4) On discharge, pertinent info about HF management at home (daily weights, low sodium diet, medications, signs and symptoms to report, and importance of keeping appointments) was reinforced. A written medication schedule was provided; (5) Outpatient follow-ups consisted of a minimum of 5 visits and 8 phone calls to assure the subjects had sufficient resources available to avoid a repeat hospitalization. The 1st home visit by the CM within the 1st two weeks of discharge. During the outpatient visits, the CM served as liaisons to the multidisciplinary team to integrate the patients' individual needs and performed a physical assessment and reviewed medications, symptom logs, diet, activity, and daily weights. Subsequent visits were conducted either in the home or clinic. The follow-up telephone calls monitored the same parameters as the visits. Interventions were based on patient need. This frequency decreased as the patients became more independent in managing their HF condition; (6) PCP and cardiologists were informed of changes in patient symptoms, and lab work was coordinated with the physicians.  Delivery personnel: Single HF specialist nurse (nurse case manager)  Communication method: Combined (Face-to-face contact and telephone calls in between meetings)  Duration & Complexity: 6 mo./multiple contacts of significant duration  Environment: Combination of settings  Com/ty: 27 | Usual care (follow-up by their PCP and a professional nurse was assigned to them each shift of each day. Care was coordinated using a primary nursing approach to patient care). | All-cause readmission  /6 mo. |
| Riegel 2002  (USA) | Multi-center  *N*=358  130 vs. 228  (Cluster RCT) | Mean age: 72.5±13.1 vs. 74.6±12.0; (73.9±12.4)  Male (%): 53.8 vs. 46.1; (48.9)  Severity of HF (%):  NYHA Ⅲ-Ⅳ: 97.6 vs. 96.4; (96.8)  LVEF: 41.9±17.0 vs. 43.2±19.1; (42.7±18.3)  Medication at discharge (%):  ACEI/ARB: 60.2 vs. 49.8  *β-*blockers: 22.8 vs. 13.4 | Intervention category: *Structured telephone support*  Recipient: Patient with some inclusion of family member  Content: (Telephonic case management provided using a decision support software program). (1) Important clinical info, individual patient needs and best practices were organized within the program to facilitate patient care by the CM. The software program used automated tools for setting priorities for patient education, data collection, and documentation; (2) Patients were telephoned within 5 d after discharge and thereafter at a frequency guided by the software and CM judgment. Patients received an average of 17 calls at decreasing intensity over the follow-up period. Each patient was estimated to have received 16 h of a CM’s time overall. Time not spent directly with patients was used in speaking with family members, consulting with community agencies and other professionals, preparing reports for physicians, and researching drugs, diets, and information requested by patients; (3) Printed educational material was mailed to patients monthly; (4) Physicians were sent automated reports produced by the software that updated them on patient progress. Guidelines for the treatment of systolic HF were distributed to physicians with their first notification of patient progress.  Delivery personnel: Single generalist nurse (registered nurse as case manager)  Communication method: Person-to-person by telephone  Duration & Complexity: 6 mo./multiple contacts  Environment: Telephone or internet-based  Com/ty: 20 | Usual care was not standardized, and no formal telephonic case-management program provided. Patients received some education regarding HF management prior to hospital discharge. | All-cause readmission  /6 mo.  HF readmission  /6 mo.  Length of hospital stay  /6 mo. |
| Riegel 2006  (USA) | Two-center  *N*=134  69 vs. 65 | Mean age: 71.6 ±10.8 vs. 72.7±11.2; (72.1±11.0)  Male (%): 42.0 vs. 40.8; (46.3)  Severity of HF (%):  NYHA Ⅲ-Ⅳ: 82.6 vs. 80.0; (81.4)  LVEF (n=105): 42.3±18.3 vs. 44.1±18.1; (43.2±18.1)  Medication at discharge (%):  ACEI/ARB: 83.4 vs. 75.0  *β-*blockers: 51.5 vs. 56.7 | Intervention category: *Structured telephone support*  Recipient: Patient with some inclusion of family member  Content: (Telephonic case management provided using a decision support software program). (1) Important clinical info, individual patient needs, and best practices were organized within the program to facilitate patient care by the CM. The software program used automated tools for setting priorities for the timing of phone call, content of patient education, data collection, and documentation; (2) Patients were telephoned within 5 d after discharge and thereafter at a frequency guided by the software and CM judgment. Patients received an average of 13.5 telephone contacts and families received an additional 8.4 telephone contacts at decreasing intensity over the follow-up period; (3) CM contacts involved consultation with other professionals (e.g., physicians, dieticians, social workers) and community agencies; (4) Printed educational material was mailed to patients monthly; (5) Physicians were sent automated reports produced by the software that updated them on patient progress.  Delivery personnel: Single generalist nurse (registered nurse as case manager)  Communication method: Person-to-person by telephone  Duration & Complexity: 6 mo./multiple contacts  Environment: Telephone or internet-based  Com/ty: 20 | Usual care was not standardised and no formal disease management programe existed at these institutions. Participants were educated regarding HF management before discharge. | All-cause readmission  /6 mo.  HF readmission  /6 mo.  Length of hospital stay  /6 mo. |
| Ritchie 2016  (USA) | Single-center  *N*=346  168 vs. 178 | Mean age: 62.7 ±12.5 vs. 63.8±13.5; (63.3±13.0)  Male (%):48.8 vs. 53.9; (51.4)  Severity of HF (%):  NYHA Ⅲ-Ⅳ: NR  LVEF: NR  Medication at discharge (%):  ACEI/ARB: NR  *β-*blockers: NR | Intervention category: *Case management*  Recipient: Patient with some inclusion of caregiver  Content: (In-hospital assessment, discretionary post-discharge support, and an interactive voice response system - supported care transition). (1) CTN assessed patient and caregiver goals using motivational interview method prior to discharge. CTN addressed relevant issues based on patient goals including medication self-management, use of a patient-centered record, primary care and specialist follow-up, and knowledge of warning signs and symptoms. The framework underlying the self-management approach focused on skill transfer to the patient with ongoing monitoring. (2) The interactive voice response system (IVR) was programmed to perform three primary functions: ⅰ. collect data from patients that would be considered transitional care “red flags” (e.g., questions about medications, escalating symptoms, insufficient clinical follow-up); ⅱ. provide customized patient education and motivation during the IVR calls based on patients’ response to questions; and ⅲ. alert the CTN when patient responses to the IVR indicated red flags. Following a practice call, participants informed the CTN what time of day would be the preferred time to receive IVR calls (less than 5 min per call). After discharge, IVR was programmed to call all patients daily for 7 d and then either daily or every 3 d, depending on patient preference, for an additional 21 calls. (3) Patients experiencing high symptom burden or significant gaps related to medication management, patient follow-up, or understanding of their overall treatment plan would trigger red flags within the system. When red flags were triggered, the CTN contacted the patient to offer additional support, based on the patient entered data. Support for patient self-management was provided through telephone-based coaching interactions when needed, up to 60 days after discharge.  Delivery personnel: Single generalist nurse (trained care transition nurses, CTN)  Communication method: Mechanized via internet or telephone  Duration & Complexity: 60 d./multiple contacts  Environment: Telephone or internet-based  Com/ty: 24 | Usual care constituted standardized discharge and postdischarge care. Floor nurses conducted discharge planning. A subset of patients received additional support from social work services or a referral for home health services. | HF readmission  /30 d. |
| Schwarz 2008  (USA) | Single-center  *N*=102  51 vs. 51 | Mean age: 77.1 ±7.3 vs. 79.1±6.9; (78.0±7.2)  Male (%): 57 vs. 39; (48.0)  Severity of HF (%):  NYHA Ⅲ-Ⅳ: 76 vs. 82; (79.4)  LVEF: NR  Medication at discharge (%):  ACEI/ARB: NR  *β-*blockers: NR | Intervention category: *Telemonitoring*  Recipient: Patient with some inclusion of caregiver  Content: (Usual care with a telemonitoring scale). (1) The patient/caregiver were provided the Cardiocom EHM system at the 1st interview within 10 d of hospital discharge, and the nurse removed the equipment 90 d later at the second interview; (2) The data collector placed a weight scale in the participants’ homes and connected via the telephone line to a computer system in the collaborating hospital. The EHM system was programmed to measure weight on a daily basis. The display on the device asked the participants to answer questions about HF symptoms, medication use, and sodium intake. The computer stored patient’s health file and automatically displayed any outliers; (3) The HF care manager, an APN, was responsible for daily monitoring of parameters and calling the caregiver to further assess the situation if necessary, provide education, and update the medication regimen; (4) The APN notified the primary physician or cardiologist about the patient’s status as needed.  Delivery personnel: Single HF specialist nurse (HF care manager)  Communication method: Mechanized via internet or telephone  Duration & Complexity: 3 mo./multiple contacts  Environment: Telephone or internet-based  Com/ty: 22 | Usual post–hospital discharge care: patients received all standard treatments and services ordered by their primary physicians/cardiologists. | All-cause readmission  /3 mo. |
| Sethares 2004  (USA) | Single-center  *N*=70  33 vs. 37 | Mean age: 75.7 ±12.3 vs. 76.8±10.5; (76.3±11.3)  Male (%): 51.5 vs. 43.2; (47.1)  Severity of HF:  NYHA Ⅲ-Ⅳ: 3.0±0.62 vs. 3.0±0.57; (3.0±0.59)  LVEF (%): 41.5±18.0 vs. 38.8±19.5; (40.1±18.7)  Medication at discharge (%):  ACEI/ARB: 57.6 vs. 64.9  *β-*blockers: 54.5 vs. 43.2 | Intervention category: *Home-visiting program*  Recipient: Patient alone  Content: (1) Subjects were interviewed during initial hospitalization using the Health Belief Scales (HBS) to determine areas in which teaching was needed; (2) Follow-up home visit within 7~10 d after hospital discharge to complete the HBS again, review medication lists, and note any medication changes; (3) Final home visit took place 1 mon after discharge to determine QoL scores and review medication changes; (4) A tailored message intervention based on the perceived benefits and barriers to HF self-care during hospitalization and 1 week and 1 mon after hospital discharge, if the person with HF scored < 4 on a benefit question or ≥3 on a barrier question. The total time for administration of the tailored message intervention averaged 15 min per participant.  Delivery personnel: Single generalist nurse (research nurse)  Communication method: Combined (Face-to-face contact)  Duration & Complexity: 1 mo./multiple contacts  Environment: Combination of settings In-patient and home-based  Com/ty: 20 | Usual care including discharge education and written educational sheets describing medications. In addition, approx. one half of the subjects also received referrals to local visiting nurse agencies. | HF readmission  /3 mo. |
| Stromberg 2003  (Sweden) | Multi-center  *N*=106  52 vs. 54 | Mean age: 77 ±7 vs. 78±6; (77.5±6.5)  Male (%): 63.5 vs. 59.3; (61.3)  Severity of HF (%):  NYHA Ⅲ-Ⅳ: 86.5 vs. 77.8; (82.1)  LVEF: NR  Medication at discharge (%):  ACEI/ARB: 86.5 vs. 77.8  *β-*blockers: 53.8 vs. 61.1 | Intervention category: *Clinic-based intervention*  Recipient: Patient with some inclusion of family member  Content: (Nurse-led HF clinic follow-up). (1) 1st clinic visit lasting 1h within 2~3 weeks after discharge, nurses evaluated status, assessed treatment and provided education about HF and social support. The individualized education included both written and verbal info, and was based on guidelines. It included info on HF, treatment, dietary advice, individually adjusted energy intake advice, lifestyle advice (including exercise), and especially aimed at promoting self-care management; (2) nurses contactable by phone during office hours, Mon~Fri, and nurses called participants to provide psychosocial support, evaluate drug changes required or other actions; (3) extra appointments to attend HF clinic scheduled for participants unstable with symptoms of worsening HF or if further education was needed; (4) participants referred back to primary health care once they were stable and well informed.  Delivery personnel: Single HF specialist nurse (specially educated and experienced cardiac nurses)  Communication method: Combined (Face-to-face contact and telephone calls in between meetings)  Duration & Complexity: unspecified/infrequent contact  Environment: Clinic/outpatient setting  Com/ty: 22 | Patients were managed in accordance with current clinical practice and received conventional follow-up in primary health care. | Length of hospital stay  /3 mo. |
| Thompson 2005  (UK) | Two-center  *N*=106  58 vs. 48  (Cluster RCT) | Mean age: 73±14 vs. 72±12; (72.5±13.1)  Male (%): 72 vs. 73; (72.6)  Severity of HF (%):  NYHA Ⅲ-Ⅳ: 76 vs. 73; (74.5)  LVEF: 31±8 vs. 29±11; (30.1±9.5)  Medication at discharge (%):  ACEI/ARB: 79 vs. 59  *β-*blockers: 25 vs. 10 | Intervention category: *Clinic-based intervention*  Recipient: Patient with some inclusion of family member  Content: (A hybrid program of clinic plus home-based intervention). (1) Appointment with specialist nurse prior to discharge, to receive info on HF and medications; (2) Office-hours contact number for nurse specialist to left messages regarding any questions or queries the patient or their family may have; (3) Home visit within 10 d of hospital discharge, for education on symptom management and lifestyle, and clinical examination; (4) Monthly nurse-led outpatient HF clinic visit for 6 mon post-discharge, including education, clinical examination and indices monitoring, and starting of new therapeutic drugs where appropriate.  Delivery personnel: Single HF specialist nurse (postgraduate qualified nurses experienced in HF management)  Communication method: Combined (Face-to-face alone and telephone calls)  Duration & Complexity: 6 mo./multiple contacts of significant duration  Environment: Combination of settings (clinic and home)  Com/ty: 27 | Standard care (i.e., explanation of their condition and prescribed medications and referral to post-discharge support as required). Patients were given an outpatient department appointment 6~8 weeks post discharge. | All-cause readmission  /6 mo. |
| Van Spall 2019  (Canada) | Multi-center  *N*=2494  1104 vs. 1390  (Stepped-wedge cluster RCT) | Mean age: 77.8±12.4 vs. 77.6±11.9; (71.7±12.1)  Male (%): 50.7 vs. 48.6; (49.6)  Severity of HF (%):  NYHA Ⅲ-Ⅳ: NR  LVEF: NR  Medication at discharge (%):  ACEI/ARB: NR  *β-*blockers: NR | Intervention category: *Case management*  Recipient: Patient with some inclusion of caregiver  Content: (Patient-Centered Care Transitions). (1) A hospital nurse navigator provided the following at the time of discharge: ⅰ. a needs assessment based on the patient’s self-reported QoL, in addition to multidisciplinary referrals (e.g., physiotherapy) as needed; ⅱ. HF self-care education to the patient and informal caregiver; ⅲ. a structured patient-centered discharge summary with a symptom-driven action plan to the patient and the family physician; ⅳ. family physician follow-up arrangements within 1 week of discharge; and ⅴ. referrals to postdischarge nurse-led home visits and HF clinic care for patients deemed high risk of readmission (assessed by LACE index concerning length of stay, acuity of presentation, comorbidities, and ED visits in the preceding 6 mon). (2) The nurse-led visits included weekly, structured, face-to-face and telephone assessments lasting 4~6 weeks until patients were seen in the HF clinic. (3) In the event of deterioration, the home-care nurse helped the patient follow the discharge action plan and contacted the HF Clinics for expedited care. (4) HF guidelines were distributed, but management was left to clinicians’ discretion.  Delivery personnel: Single generalist nurse (a hospital nurse navigator)  Communication method: Combined (Face-to-face contact and telephone calls)  Duration & Complexity: 3 mo./multiple contacts of significant duration  Environment: Combination of settings  Com/ty: 27 | Transitional care occurred at the discretion of clinicians. In 1 hospital, a nurse provided education and a home visit to select patients. Eight participating hospitals had access to regional HF clinics, while 2 did not. | All-cause readmission  /3 mo.  Emergency department visit  /3 mo. |
| ^*^ Intervention versus Control (Total) unless otherwise specified.  ^†^ NYHA, New York Heart Association. The percentage of participants with NYHA Class Ⅲ-Ⅳ are only presented.  ^‡^ LVEF, Left Ventricular Ejection Fraction.  ^§^ Overall intensity and complexity rated using the revised HF Disease Management Scoring Instrument (HF-DMSI).  ^¶^ Timing of outcome measurement. | | | | | |
