# Supplementary material for: Effects of nurse-led transitional care interventions for patients with heart failure on healthcare utilization: A meta-analysis of randomized controlled trials
Source: PLoS One. 2021 Dec 16;16(12):e0261300. doi: 10.1371/journal.pone.0261300 (PMC8675680; doi:10.1371/journal.pone.0261300)

## Sensitivity analysis

# All-cause readmissions

- Sensitivity analysis
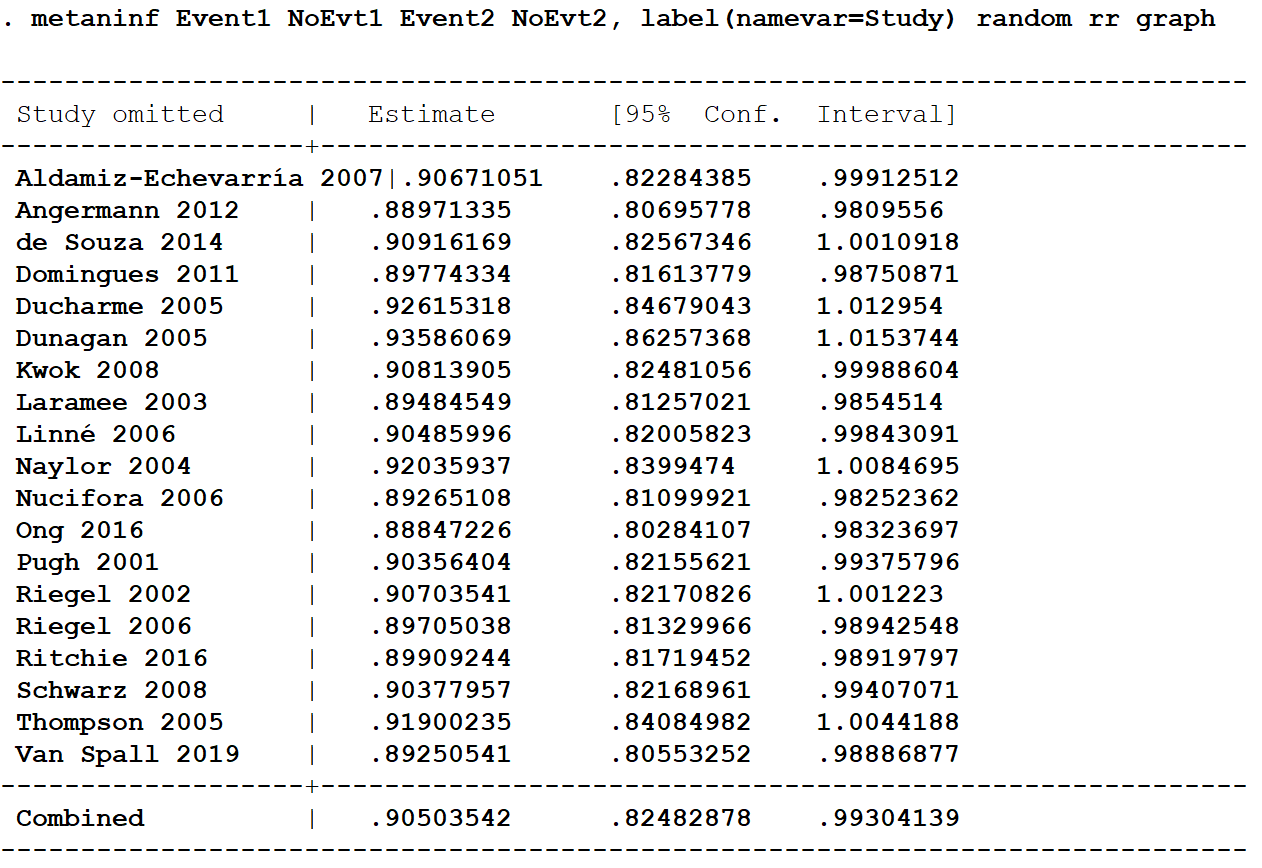


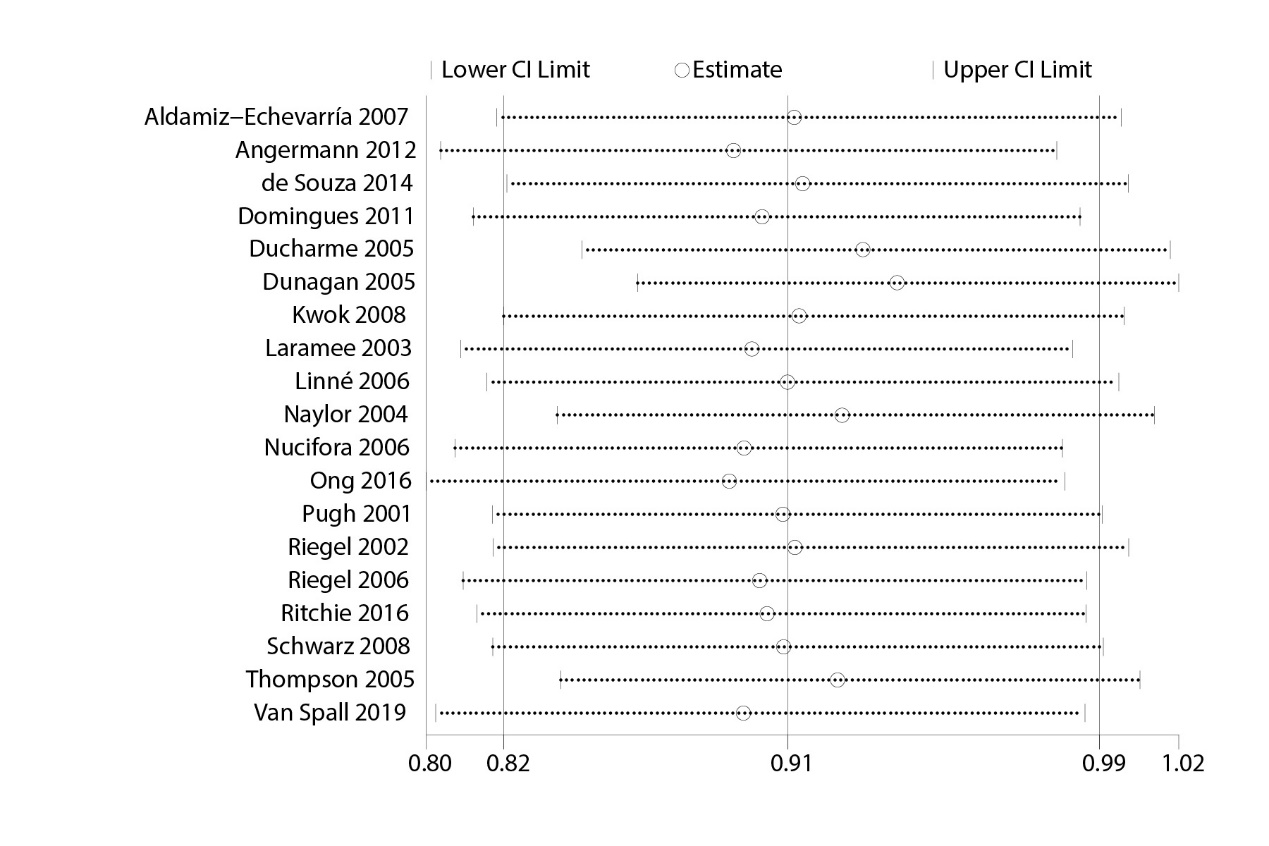


# HF-specific readmissions

## Sensitivity analysis


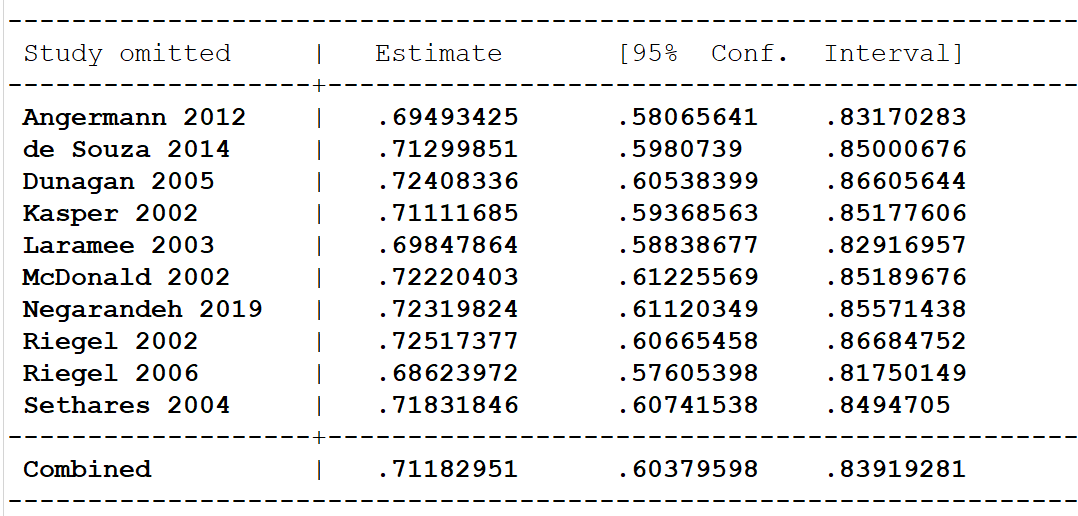


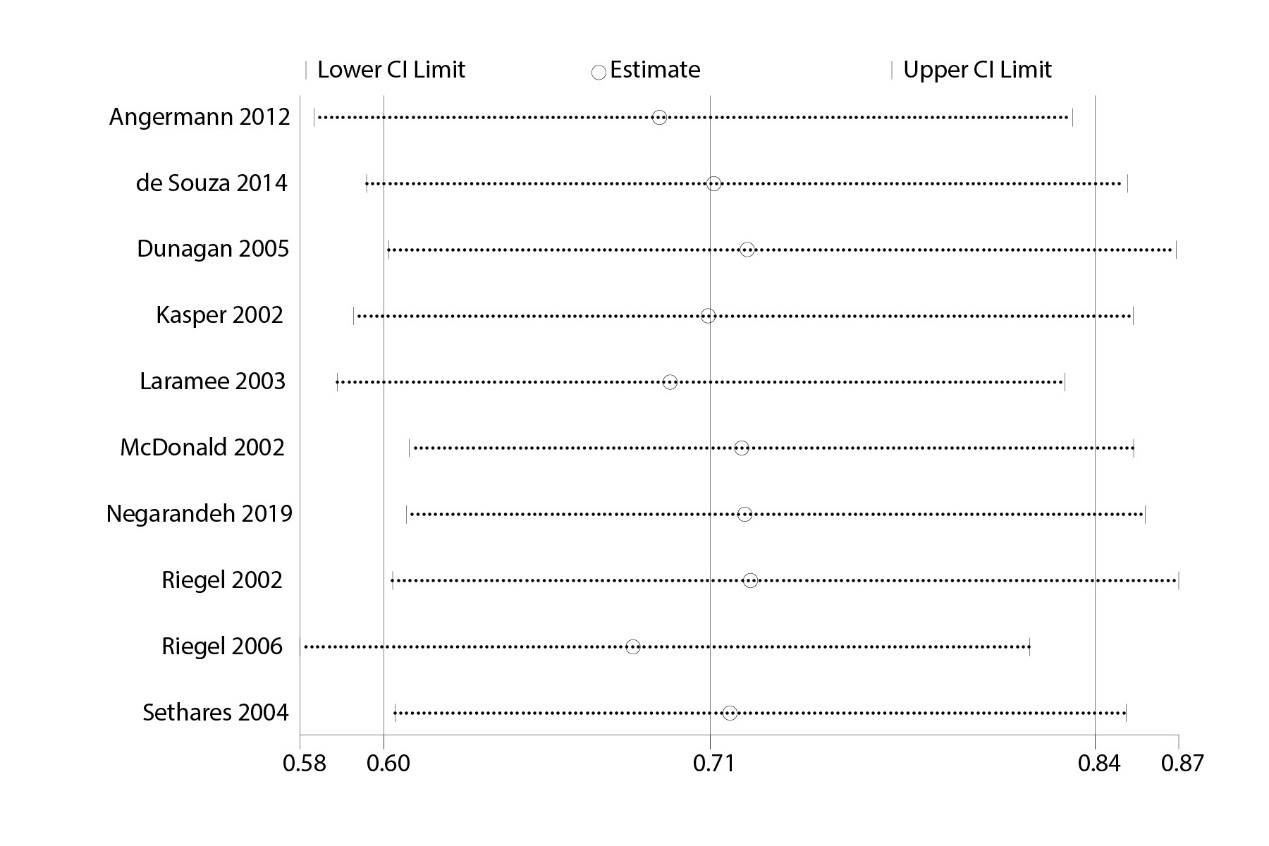


# Emergency department visits

## Sensitivity analysis


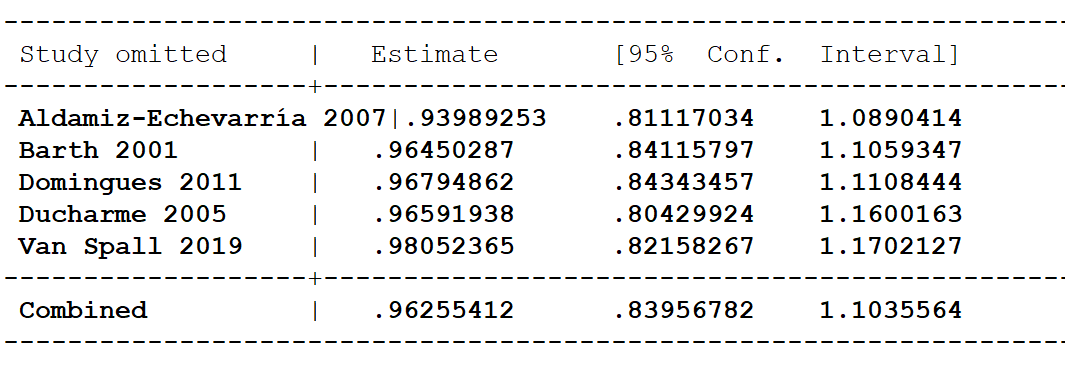


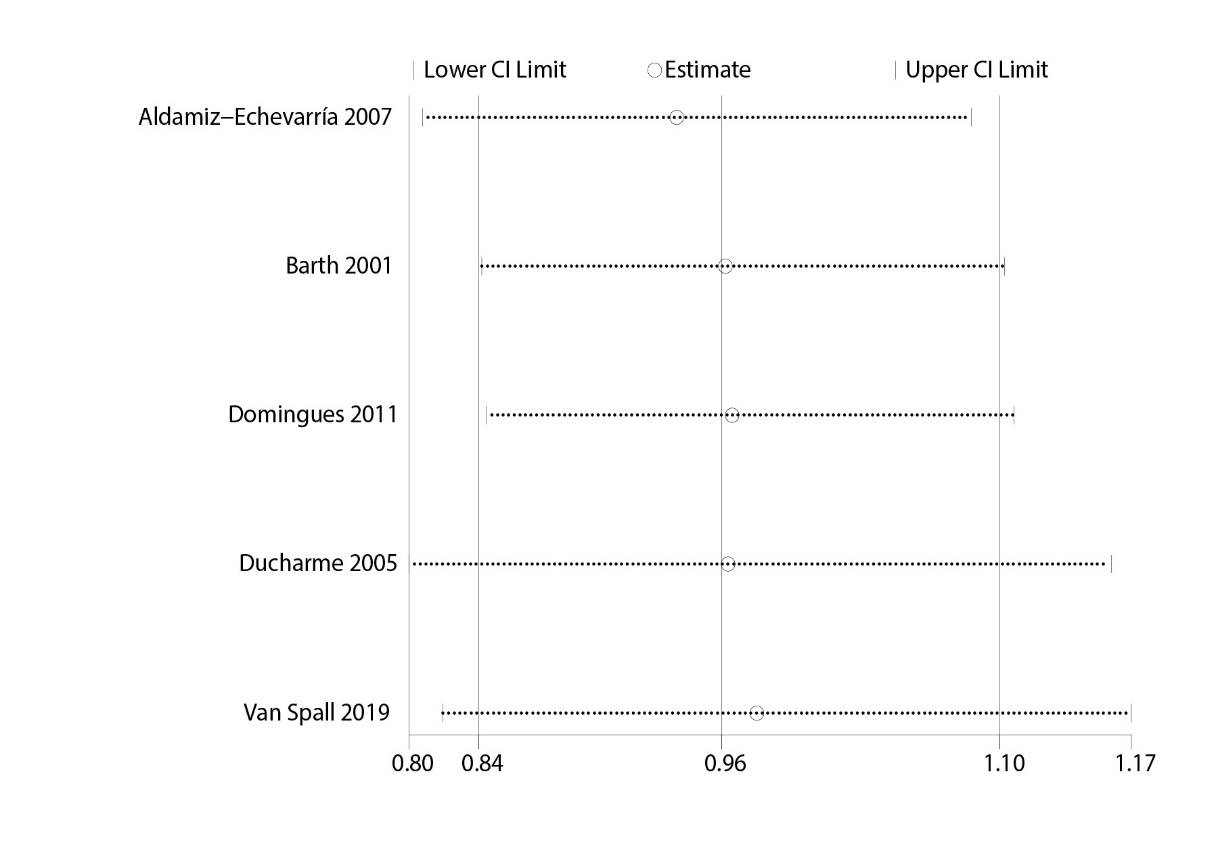


# Length of hospital stay

## Sensitivity analysis


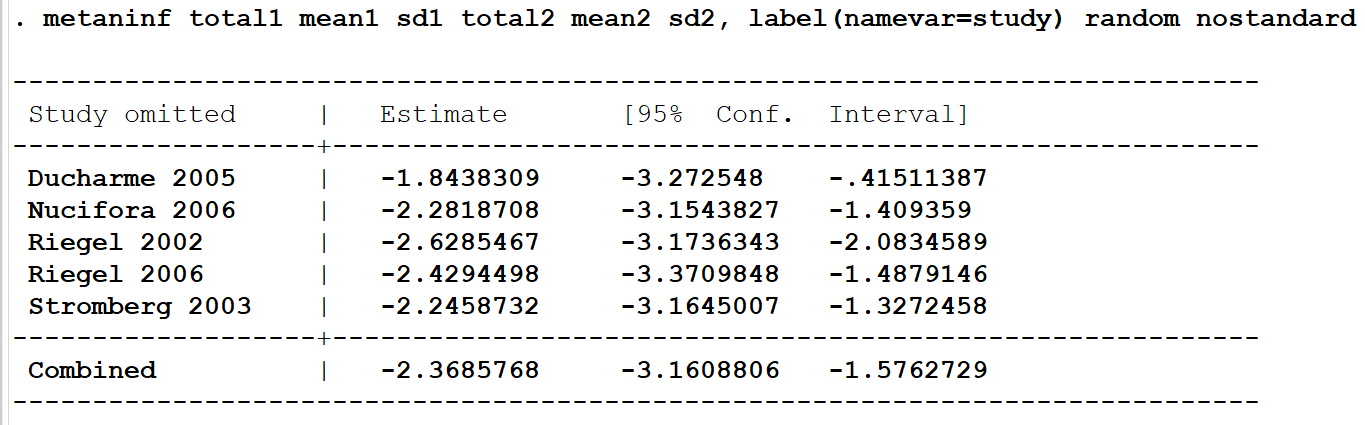


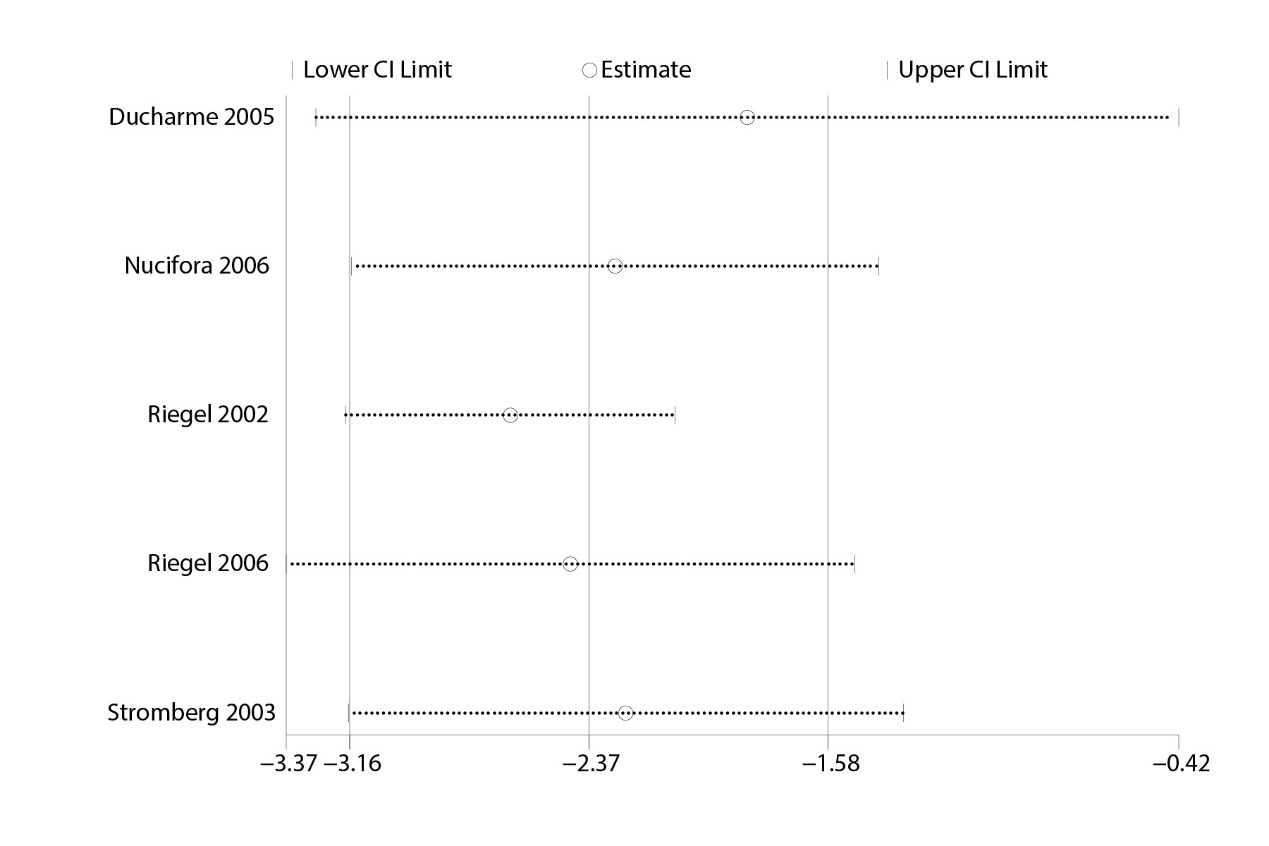

Supplement: S4 File — (DOCX) [file pone.0261300.s005.docx]
