# Supplementary material for: Effects of nurse-led transitional care interventions for patients with heart failure on healthcare utilization: A meta-analysis of randomized controlled trials
Source: PLoS One. 2021 Dec 16;16(12):e0261300. doi: 10.1371/journal.pone.0261300 (PMC8675680; doi:10.1371/journal.pone.0261300)
Supplement: S5 File — (DOCX) [file pone.0261300.s006.docx]

## Univariate meta-regression analysis of all-cause and HF-specific readmissions

|  | All-cause readmissions | | | HF-specific readmissions | | |
| --- | --- | --- | --- | --- | --- | --- |
|  | No. | β (95% CI) | p Value | No. | β (95% CI) | p Value |
| *Trial* *Characteristics* |  |  |  |  |  |  |
| Study location ^*^ | 19 | -0.035 (-0.426, 0.356) | 0.85 | 10 | 0.136 (-0.411, 0.682) | 0.58 |
| Year of publication | 19 | 0.014 (-0.001, 0.030) | 0.07 | 10 | -0.002 (-0.042, 0.037) | 0.91 |
| Study quality ^†^ |  |  |  |  |  |  |
| Low risk of bias | 5 | — | — | 1 | — | — |
| Some concerns | 9 | -0.121 (-0.358, 0.116) | 0.30 | 6 | -0.119 (-0.672, 0.435) | 0.63 |
| High risk of bias | 5 | 0.040 (-0.276, 0.356) | 0.79 | 3 | 0.295 (-1.014, 0.425) | 0.37 |
| *Participant characteristics* |  |  |  |  |  |  |
| Mean age | 19 | -0.008 (-0.034, 0.018) | 0.55 | 9 | -0.003 (-0.048, 0.041) | 0.86 |
| Percentage of men | 18 | 0.001 (-0.009, 0.012) | 0.08 | 10 | 0.001 (-0.019, 0.022) | 0.91 |
| LVEF ^‡^ | 15 | -0.177 (-0.432, 0.079) | 0.16 | 9 | -0.014 (-0.459, 0.430) | 0.94 |
| NYHA ^§^ | 12 | -0.007 (-0.014, 0.000) | 0.06 | 7 | -0.003 (-0.014, 0.008) | 0.51 |
| *Intervention characteristics* |  |  |  |  |  |  |
| Delivery personnel |  |  |  |  |  |  |
| Single generalist provider | 11 | — | — | 5 | — | — |
| Single HF expert provider | 6 | -0.054 (-0.318, 0.210) | 0.67 | 2 | 0.128 (-0.440, 0.697) | 0.61 |
| Multidisciplinary team | 2 | -0.035 (-0.368, 0.298) | 0.83 | 3 | 0.063(-0.410, 0.536) | 0.76 |
| Communication method |  |  |  |  |  |  |
| Internet/Telephone-mechanized | 1 | — | — | 0 | — | — |
| Person-to-person by telephone | 6 | 0.017 (-0.853, 0.888) | 0.97 | 5 | — | — |
| Face-to-face communication | 2 | -0.096 (-1.027, 0.835) | 0.83 | 0 | — | — |
| Combined methods | 9 | -0.050 (-0.918, 0.818) | 0.90 | 5 | -0.030 (-0.430, 0.369) | 0.87 |
| Intervention environment |  |  |  |  |  |  |
| Inpatient | 1 | — | — | 0 | — | — |
| Telephone or internet-based | 8 | 0.070 (-0.416, 0.557) | 0.76 | 5 | — | — |
| Home-based | 1 | -0.036 (-0.713, 0.649) | 0.91 | 1 | 0.250 (-1.373, 0.872) | 0.61 |
| Combination of settings | 9 | -0.030 (-0.516, 0.456) | 0.90 | 4 | -0.007 (-0.452, 0.438) | 0.97 |
| Involvement of caregiver | 19 | 0.006 (-0.224, 0.238) | 0.95 | 10 | 0.160 (-0.238, 0.558) | 0.38 |
| Place of intervention initiated ^¶^ | 19 | 0.179 (-0.011, 0.368) | 0.06 | 10 | -0.003 (-0.417, 0.411) | 0.99 |
| Intervention length | 19 | -0.023 (-0.077, 0.032) | 0.39 | 10 | 0.048 (-0.089, 0.185) | 0.45 |
| ^*^ Study location grouped according to country income classifications for the World Bank’s 2020.  ^†^ Study quality assessed using the latest version of the Cochrane risk-of-bias tool for randomized trials (RoB 2).  ^‡^ LVEF, Left Ventricular Ejection Fraction.  ^§^ NYHA, New York Heart Association functional classification.  ^¶^ Place of intervention initiated in hospital or home. | | | | | | |
