# Supplementary material for: Effects of nurse-led transitional care interventions for patients with heart failure on healthcare utilization: A meta-analysis of randomized controlled trials
Source: PLoS One. 2021 Dec 16;16(12):e0261300. doi: 10.1371/journal.pone.0261300 (PMC8675680; doi:10.1371/journal.pone.0261300)
Supplement: S6 File — Funnel plots for the effect of nurse-led TCIs on (a) all-cause readmissions and (b) HF-specific readmissions. (DOCX) [file pone.0261300.s007.docx]

## Funnel plots for the effect of the nurse-led TCIs on (a) all-cause

**readmissions and (b) HF-specific readmissions**

**
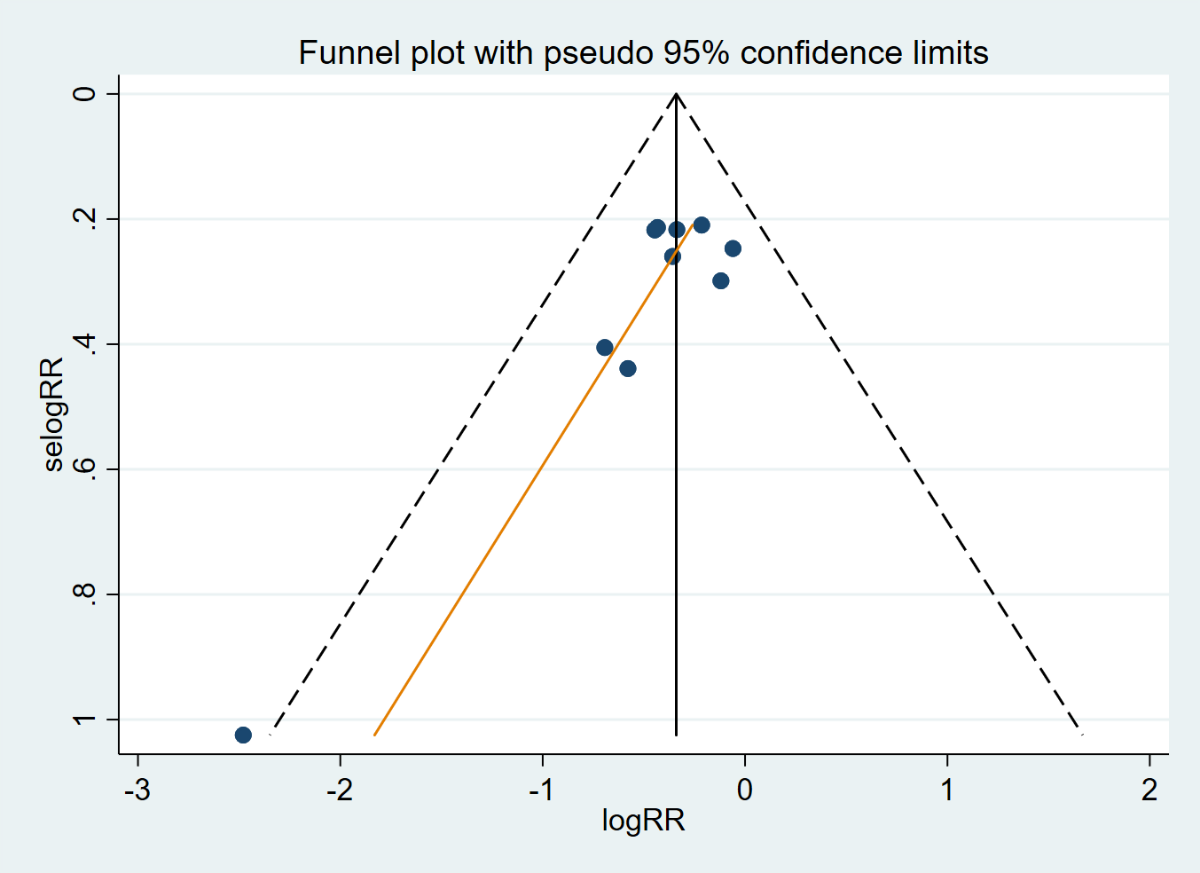
a
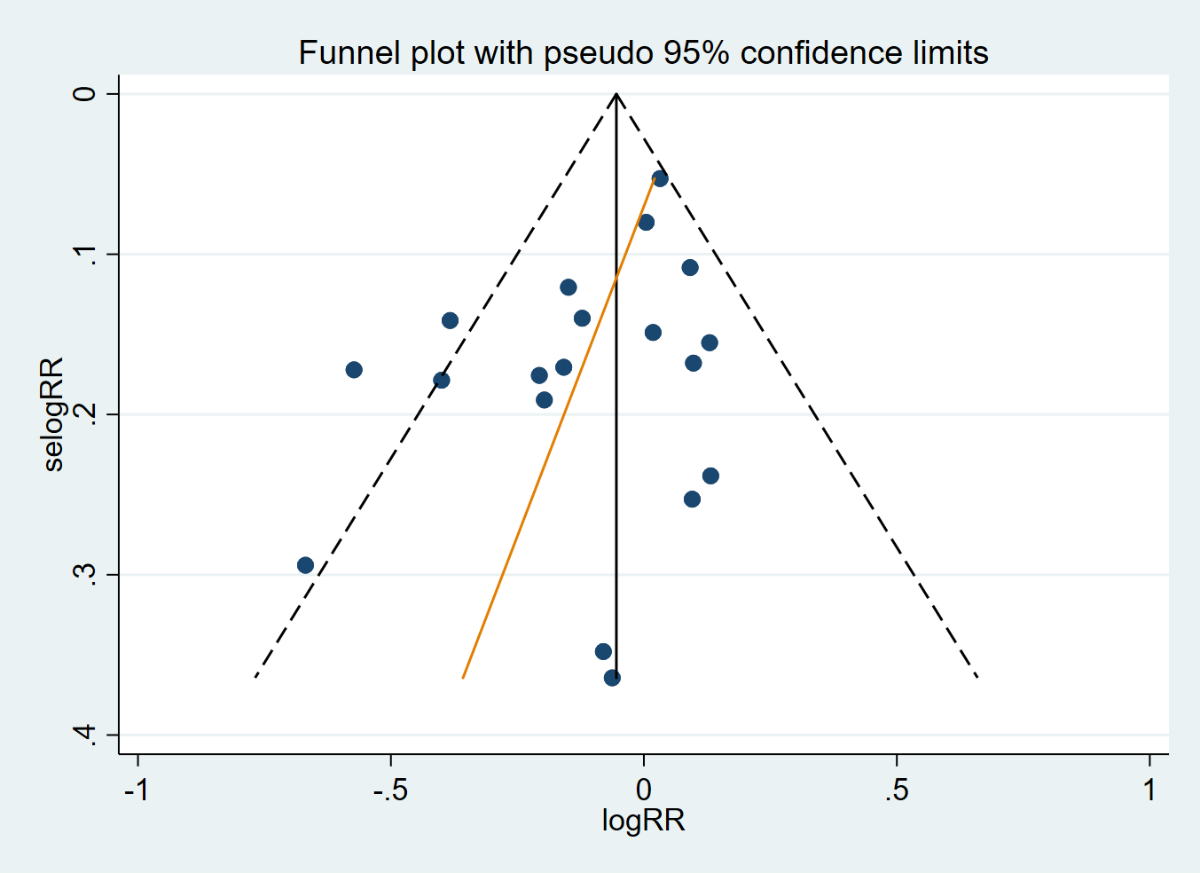
**

**b**
