# Supplementary material for: Effects of nurse-led transitional care interventions for patients with heart failure on healthcare utilization: A meta-analysis of randomized controlled trials
Source: PLoS One. 2021 Dec 16;16(12):e0261300. doi: 10.1371/journal.pone.0261300 (PMC8675680; doi:10.1371/journal.pone.0261300)
Supplement: S7 File — (DOCX) [file pone.0261300.s008.docx]

## Summary of findings

**Nurse-led transitional care intervention compared to usual care for patients with heart failure**

**Patients:** Adults with heart failure

**Setting**: Home / community / outpatient

**Intervention:** Nurse-led transitional care intervention

**Comparison:** Usual care

| **Outcomes** | **Risk of Bias** | **Inconsistency** | **Indirectness** | **Imprecision** | **Other**  **Considerations** | **№ of patients (studies)** | **Relative effects**  **(95% CI)** | **Certainty of the evidence**  **(GRADE)** | **Comments** |
| --- | --- | --- | --- | --- | --- | --- | --- | --- | --- |
| **All-cause readmission** | serious ^a^ | serious ^b^ | not serious | not serious | none | 7834  (19 RCTs) | **RR 0.91** (0.82 to 0.99) | ⨁⨁◯◯  LOW |  |
| **HF readmission** | very serious ^a c^ | not serious | not serious | not serious | dose response gradient ^d^ | 2345  (10 RCTs) | **RR 0.71** (0.60 to 0.84) | ⨁⨁⨁◯  MODERATE |  |
| **Emergency department visit** | serious ^c^ | not serious | not serious | serious ^e^ | none | 3148  (5 RCTs) | **RR 0.96** (0.84 to 1.10) | ⨁⨁◯◯  LOW |  |
| **Length of hospital stay** | serious ^a^ | not serious | not serious | serious ^e^ | none | 1028  (5 RCTs) | **RR -2.37** (-3.16 to -1.58) | ⨁⨁◯◯  LOW |  |

**CI:** Confidence interval; **RR:** Risk ratio

**GRADE Working Group grades of evidence**

**High certainty:** We are very confident that the true effect lies close to that of the estimate of the effect.

**Moderate certainty:** We are moderately confident in the effect estimate: The true effect is likely to be close to the estimate of the effect, but there is a possibility that it is substantially different.

**Low certainty:** Our confidence in the effect estimate is limited: The true effect may be substantially different from the estimate of the effect.

**Very low certainty:** We have very little confidence in the effect estimate: The true effect is likely to be substantially different from the estimate of effect.

1. Downgraded by one level due to lack of adequately blinding introduced bias.
2. Downgraded by one level due to unexplained inconsistency (some heterogeneity I^2^=46%, p=0.01).
3. Downgraded by one level due to the high risk of bias (one studies may have selective reporting results).
4. Upgraded by one level due to a linear relationship between the scores of the adapted HF-DMSI and HF readmissions.
5. Downgraded by one level due to serious imprecision.
