# Supplementary material for: Effects of nurse-led transitional care interventions for patients with heart failure on healthcare utilization: A meta-analysis of randomized controlled trials
Source: PLoS One. 2021 Dec 16;16(12):e0261300. doi: 10.1371/journal.pone.0261300 (PMC8675680; doi:10.1371/journal.pone.0261300)
Supplement: Attachment — Submitted filename: Response to Reviewers.docx [file pone.0261300.s010.docx]

Dear editor and reviewers,

Thank you for your letter dated 6 October 2021.

On behalf of my co-authors, I am herewith submitting the revised manuscript entitled “Effects of nurse-led transitional care interventions for patients with heart failure on healthcare utilization: A meta-analysis of randomized controlled trials.” (PONE-D-21-01523_R2) for consideration of publication in PLOS ONE. We would like to thank the editor and reviewers’ work devoted to our manuscript and we are very grateful for their valuable suggestions. We have considered the comments carefully and have made revisions (highlighted in red in the revised manuscript) which we hope meet with approval.

**Comment to the Author:**

**Reviewer #2:**

1. “I appreciate the changes made by the authors in response to reviewer comments. With regards to the adapted HF-DMSI, Table 1 should not use in its last column header "COM/Ty" and instead use "Adapted HF-DMSI" to be consistent with the text of the manuscript. I would recommend changing the Table 1 footnote by taking out the first two words "COM/Ty: the" and capitalize "adapted" so that the footnote helps a reader understand how the HF-DMSI was adapted (and can look for more information in the text and supplement).”

**Response:** We appreciate the time the Reviewer #2 spent in making the constructive suggestions. We have revised the last column header and the footnote in the table 1 in the revised manuscript as suggested.

We hope the revised manuscript is now suitable for publication in PLOS ONE and look forward to hearing from you soon.

Kindest regards,

Jinbo Fang
